# Screen and Triage by Community Extension Workers to Facilitate Screen and Treat: Task-Sharing Strategy to Achieve Universal Coverage for Cervical Cancer Screening in Nigeria

**DOI:** 10.1200/JGO.18.00023

**Published:** 2018-07-26

**Authors:** Olutosin A. Awolude, Sunday O. Oyerinde, Joshua O. Akinyemi

**Affiliations:** **Olutosin A. Awolude**, **Joshua O. Akinyemi**, College of Medicine, University of Ibadan; **Olutosin A. Awolude** and **Sunday O. Oyerinde**, University College Hospital, Ibadan, Nigeria.

## Abstract

**Purpose:**

Universal coverage of cervical cancer screening remains elusive in most low- and middle-income countries (LMICs), home to the greatest burden of this preventable disease. Implementation of a cytology-based screening strategy in these countries is challenging. Also, there is shortage of health care workers (HCWs) to implement the low-technology, cheaper, but equally effective, methods like visual inspection with acetic acid. However, the implementation of HIV programs in LMICs has introduced the innovation of task shifting and task sharing, using the community health extension workers (CHEWs) and community health officers (CHOs) to complement clinical HCWs, especially at the primary health care, level with good outcome. Hence, this study leveraged this strategy.

**Methods:**

We piloted a study to improve knowledge and practice skills of CHEWs and CHOs in a rural community of Oyo state, Nigeria, through training and participatory supervision to screen for cervical cancer using visual inspection with acetic acid and link positive cases for treatment with cryotherapy.

**Results:**

A total of 51 HCWs, including doctors, nurses, CHEWs, and CHOs, were trained during the study to provide cervical cancer screening services. After the training, cervical cancer and its prevention knowledge improved from 52.4% before training to 91.5% immediate after training. Over 12 months, 950 eligible women were screened, of whom 848 (89.3%) were screened by CHEWs and CHOs. Of the 63 rescreened by CHEWs and CHOs (data grouped), and nurses, 88.1% and 92.3%, respectively, agreed with expert team review, with κ statistics of 0.76 and 0.84, respectively.

**Conclusion:**

This pilot project showed the ability of CHEWs and CHOs to identify cervical dysplasia was good and that of nurses was very good with appropriate competency training to achieve universal coverage of cervical cancer screening in LMICs.

## INTRODUCTION

Effective screening programs for precancer and cancerous lesions of the cervix can lead to reduced morbidity and mortality from this preventable condition.^1,2^ In low- and middle-income countries (LMICs), there is often lack of access to effective screening for cervical cancer, leading to high cause-related rates of morbidity and mortality. This is particularly the case in populations in rural areas where health care access is characterized by transport challenges, ill-equipped health facilities, lack of information access, and disproportionately lower numbers of highly trained HCWs.^3^ Approximately one-half of cervical cancers occur in women who have never been screened,^4^ many of whom are poor, reside in rural areas, and lack access to health care workers (HCWs) and infrastructure.^5^

In high-resource settings, cervical cytology screening programs have successfully reduced the rates of cervical cancer.^6^ But in many low-resource settings, few women have access to cytology screening programs, due to the absence of functional national screening programs and lack of equipment and skilled technicians.^7^ As a result, other low-cost methods, like visual inspection with acetic acid (VIA), have been implemented for screening by trained nurses and doctors.^8,9^ Even so, many of these highly trained HCWs are largely absent in rural communities in developing countries like Nigeria, where > 70% of the population at risk for cervical cancer reside. In 2006, the World Health Organization (WHO) recognized a shortage of HCWs and the associated decreased access to care. This is especially so in sub-Saharan Africa.

However, the implementation of HIV programs in sub-Saharan Africa has introduced the innovation of task shifting and task sharing using the community health extension workers (CHEWs) to complement the workforce, especially at primary health care (PHC) centers and in rural communities with good outcome both in coverage and quality of service delivery. Task shifting refers to transferring tasks to HCWs who have not conventionally performed these tasks as part of their scope of practice.^2,10^ The workers to whom tasks are shifted are more readily available, completed shorter training, and have fewer qualifications.^11^ Task sharing emphasizes a knowledge-based requirement for delegated roles and responsibilities. Today, task shifting, and task sharing have been used in more than HIV services and practiced in almost all health facilities, either formally or informally, across a number of disciplines, including midwifery, surgery, and noncommunicable disease management.^12-18^

The objectives of this study were to evaluate the effects of a program of short-term, competency-based cervical cancer screening training of HCWs in primary and secondary health care levels of a rural local government of Nigeria and to determine if CHEW- or CHO-led VIA screening for cervical cancer in a low-resource setting had similar results to that of a nurse-led screening in detecting cervical lesions as a task-sharing strategy to facilitate universal coverage of cervical cancer screening in our setting. We hypothesized that intensive, applied training with supportive supervision would enhance the abilities of these less trained HCWs to provide quality cervical cancer screening services and increase population coverage of the service in the local government area.

## METHODOLOGY

### Study Design

This was a cross-sectional comparative study evaluating the accuracy of cervical cancer screening using VIA method by CHEWs and CHOs, whose data were grouped, compared with nurses in detecting cervical lesions. This rural community-level interventional strategy involved identifying and enhancing skills of CHEWs, CHOs, and nurses in PHCs and a general hospital in a rural local government area. The HCWs had training, technical and infrastructural supports, and participatory supervision. This study was approved by the ethics committees of the University of Ibadan/University College Hospital, Ibadan, and Oyo State Ministry of Health.

### Study Participants

#### Training of participants

The CHEWs, CHOs, nurse-midwives, and medical officers working in the PHCs and the general hospital in the Ibarapa central local government area of Oyo State were trained on the principles and practice of VIA as a screening modality for premalignant lesions of the cervix by three of the five members of the study team: a consultant gynecologist, a senior resident in obstetrics and gynecology, and a cytology nurse. In addition, the medical officer and the nurses had training on the use of cryotherapy to enable them to treat eligible patients after screening.

The training had two components. The first was competency-based training combining didactic lectures, picture training of normal and abnormal VIA test results, and classroom pelvic model practice for 1.5 days, and 3.5 days of clinic-based hands-on training performing and interpreting VIA tests, the appropriate referral system to the general hospital that served as the hub for the PHCs, and appropriate evaluation and treatment of cases of cervical lesions using cryotherapy for eligible cases and referral for other cases. Each participant examined, on the average, between three and five patients during the practical training, because we did not have a prespecified minimum number. The second was onsite continuation of participatory practical training after 2 weeks of the initial training and one to two monthly supervisory site visits throughout the duration of the pilot study. The didactic lecture topics included normal anatomy and physiology of the vulva, vagina, and cervix; abnormal vagina and cervix; screening to detect the precursors of cervical cancer (including using appropriate pictures); counseling and informed choice; VIA; recording findings and safety issues; principles of infection prevention; and appropriate documentation and referral systems. The classroom practical sessions covered techniques of wearing gloves, patient preparation and positioning, insertion of a vaginal speculum with identification of cervix using a pelvic model, technique of visual inspection of the cervix, pictures of normal and abnormal cervices, technique for obtaining a sample for the Papanicolaou test, technique of application of acetic acid, and post-test counseling of patients.

#### Cervical cancer screening participants

Using the infrastructure already established through the roll out of the President's Emergency Plan for AIDS Relief–funded HIV care and treatment program, coordinated by our institution, the trained participating HCWs counseled and provided cervical cancer screening services to outpatients who were seen for for other nonemergency conditions in five of the 11 suitable PHCs and the only general hospital in the local government area, and who gave verbal informed consent.

### Evaluation of Activities

Participating HCWs’ knowledge of cervical precancer and cancerous lesions, causes, prevention and treatment options, and ability to recognize normal and abnormal cervices were evaluated before and after the training intervention, using a self-administered interview survey and picture tests. Items in the survey instrument included sociodemographic variables such as age, sex, marital status, highest educational qualification, professional category, as well as questions evaluating knowledge of cervical precancerous and cancerous lesions, the cause, risk factors, and prevention. The instrument for the self-administered interview was designed by the study team. It was piloted during similar training for a nongovernmental organization in a town about 200 km from this local government area a year before this study, followed by refinement of ambiguous or deletion of redundant questions before preparation of the final version of the questionnaire. For the current study, we added the picture test to the training on theory, and technique of wearing gloves to the classroom practical training. The improvement in knowledge was assessed by comparing the participants’ pretraining and post-training performances on the survey questions and the picture test.

#### Intervention activities for comparison of competency between CHEWs, CHOs, nurses, and midwives

All the participating women initially underwent VIA screening for cervical precancerous lesions by either the CHEWs and CHOs in PHCs or the nurses in the general hospital. All patients with positive screening results by VIA and 10% of age-paired women whose VIA screening results were negative were recalled for rescreening and treatment using a participatory supervision approach after informed consent was obtained. During this period, each of the recalled participants had a self-held, nonlubricated speculum placed in the vagina, and the cervix was visualized for any obvious lesion or significant cervical mucus, which is gently wiped off with cotton wool on a sponge-holding forceps, if present. A Papanicolaou test sample was then collected. The cervix was wiped with 5% acetic acid for 1 minute, followed by evaluation for aceto-whitening, margins, and surface and lesion size by the CHEWs or CHOs from the PHC where the woman was previously screened, followed by examination by the nurse from the general hospital under the guidance of a member of the supervisory team consisting of a gynecologist, a resident doctor, and cervical cytology nurse/trainer, with the reports agreed on. For women previously screened in the general hospital, the nurse performed the first evaluation. The results from the examinations were reported by the CHEWs, CHOs, and the nurses to the study coordinator in a separate location. The CHEWs, CHOs, and the nurses were not allowed to communicate the patient’s examination results among themselves.

Women for whom VIA results were positive, as agreed, and eligible, underwent cryotherapy treatment. They were appropriately provided with postcryotherapy counseling on follow-up, procedure-related danger signs, and follow-up schedule screening for evidence of cure 1 year later. Women for whom VIA results were positive but who were not amenable to cryotherapy treatment and patients identified with invasive cervical cancer underwent cervical biopsy done by the gynecologist or the resident doctor. Such samples, as well as the Papanicolaou test samples, were processed in Pathology Department of our institution. The eligible patients were referred for loop electrical-excision procedure and radiotherapy treatment in the teaching hospital (results not shown).

### Data Analysis

To ascertain the comparability of the participants who took the pretest and post-test, we compared their sociodemographic characteristics using the independent *t* test and χ^2^ test for age and categorical variables, respectively. We explored cervical precancer and cancer, prevention, and treatment knowledge before and after training. To test the level of agreement between the CHEWs- and CHOs-led and nurse- and midwife-led cervical cancer screening, the percentage agreement and the κ statistic were calculated. The κ statistics were graded as poor (≤ 0.60), good (0.61 to 0.80), and very good (0.81 to 1.00), on the basis of a recommendation by Landis and Koch.^19^

## RESULTS

### Training Activities

**Fig 1:**
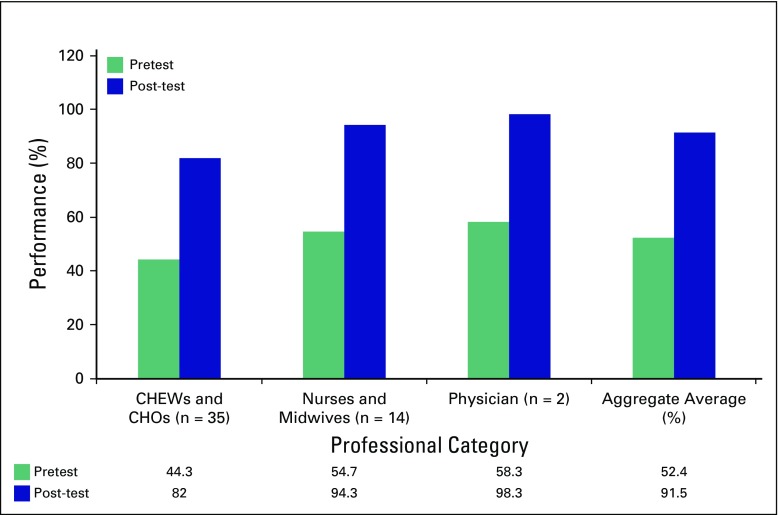
Effect of the training on participants’ knowledge by professional category. CHEW, community health extension worker; CHO, community health officer.

During the study, a total of 51 HCWs were trained: two physicians (3.9%), 12 nurses or midwives (23.5%), and 37 CHEWs or CHOs (72.6%). Table 1 lists the sociodemographic characteristics of the participants who completed the pre- and postworkshop surveys, and these were similar with respect to age, sex, and work category. Figure 1 shows the effect of the training program on participants' pre- and post-training knowledge of cervical precancer and cancer causes, prevention and treatment types, as well as the ability to recognize pictures of normal and abnormal cervices and possible results of VIA test results. On average, knowledge about cervical cancer, identification of normal and abnormal cervices, and knowledge of screening and treatment options for precancerous lesions improved from 52.4% pretraining (CHEWs and CHOs, 44.3%; nurses and midwives, 54.7%; physicians, 58.3%) to 91.5% immediately after training (CHEWs and CHOs, 82.0%; nurses and midwives, 94.3%; physician, 98.3%).

**Table 1:**
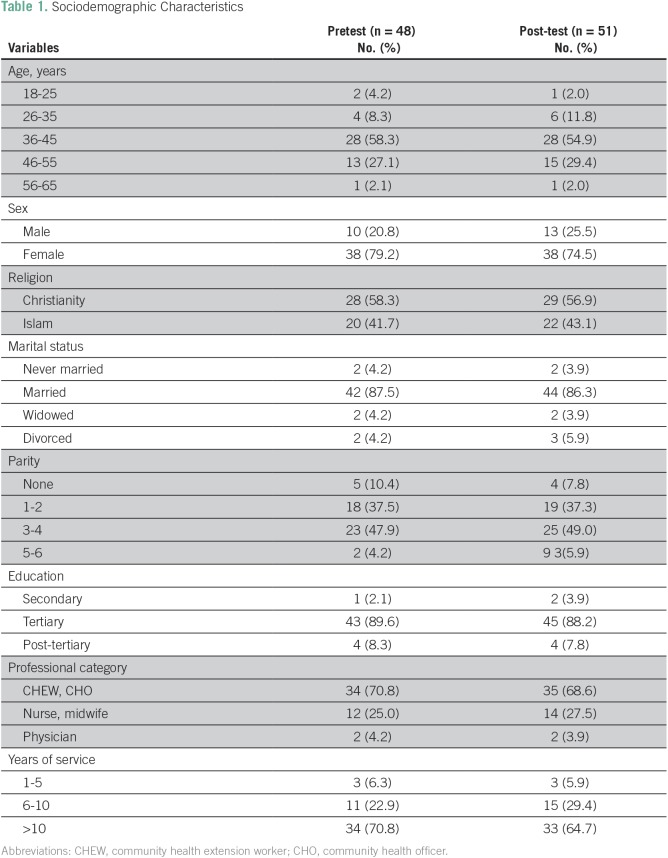
Sociodemographic Characteristics

### Intervention Activities

A total of 950 women were screened for cervical cancer over 1 year, of whom 848 (89.3%) were screened in five PHCs by CHEWs and 102 (10.7%) in a general hospital by nurses. A total of 66 cases (6.9%) were identified as VIA positive (81.8% of cases from PHCs and 18.2% from the hospital). These 66 women with VIA-positive results and 88 women with negative VIA results were recalled for rescreening. Of these, 143 (63 VIA positive; 80 VIA negative) returned for follow-up assessment. The mean age of these women was 35.8 (SD, 13.2) years. Their parity ranged from zero to seven, with a modal parity of three. Approximately two-thirds of the women (63.7%) were not using any contraceptive method. Among those who did use contraception, the commonest methods were the intrauterine contraceptive device (16.4%) and depot medroxyprogesterone acetate (11.6%).

Results of follow-up assessment by CHEWs and CHOs as a group, nurses, and the expert assessor are presented in Table 2 along with the statistics on interrater agreement. Percentage agreement by CHEWs and CHOs as a group, and nurses were 88.1% and 92.3%, respectively, with a κ value for CHEWs and CHOS of 0.76, and κ value for nurses of 0.84, implying good and very good agreement, respectively. However, true-positive rates were higher among nurses (93.5%) compared with CHEWs and CHOs (82.6%), and false-positive rates were 14.8% and 8.6%, respectively. Of the 63 Papanicolaou test samples, 57 (90.5%) were adequate for reporting and 48 (84.2%) of these showed dysplastic lesions of low- and high-grade intraepithelial lesions, six (10.5%) had inflammatory lesions, and three (5.3%) were negative for intraepithelial lesions.

**Table 2:**
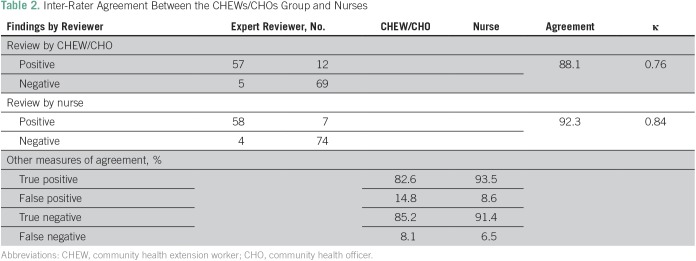
Inter-Rater Agreement Between the CHEWs/CHOs Group and Nurses

## DISCUSSION

HCWs with less training, like CHEWs and CHOs, are being used increasingly in countries, especially in LMICs, to perform health care responsibilities otherwise not within their professional schedule, using task-shifting and task-sharing principles. Task-shifting strategy is a rational distribution of primary care duties from physicians to nonphysician HCWs.^10,20^ The more recent term of task sharing emphasizes a knowledge-based requirement for delegated roles and responsibilities.^21^ Whereas task shifting focuses on the delegation of duties, task sharing incorporates collective input of the health unit or health team. Task-shifting strategies have the potential to mitigate the systems-level barriers to implementation of primary and secondary prevention. This is particularly important in LMICs in the areas of disease prevention, health promotion, treatment support, counseling, and home-based care.^22^ As most community HCWs live in the communities they serve, challenges of cultural and language competence are mitigated.^23^

In task-shifting and task-sharing processes, many enablers of success have been identified, including health system factors such as training^24^ and provision of guidelines for screening^25-29^ with significant changes in the knowledge level of HCWs reported.^30,31^ In one study, the knowledge regarding the choice of correct antihypertensive drugs improved substantially after training from 17% to 94%^30^; in another study, results indicated that the knowledge levels of nonphysician HCWs trained in the management of cerebrovascular disease increased from 47% to 93% after a 4-day training program.^30^ In our study, we found that using a short-term, multimodal task-sharing training of 5 days and ongoing supportive supervision intervention, knowledge of risk factors for cervical cancer and precancer screening methods significantly improved from 52.4% to 91.5% among this cohort of CHEWs, CHOs, and other HCWs trained in this pilot local government area. The improved cervical cancer prevention knowledge among these HCWs, most of whom were CHEWs or CHOs, empowers them in early detection of cases of precancerous lesions of the cervix at PHCs, which have minimal health services. This improvement in knowledge and skills enhanced linkage with the higher-level health facilities where the patients had access to the needed cryotherapy treatment with implications for the expected positive outcome.^32^ Furthermore, closing the cervical cancer prevention knowledge gap of HCWs enhances their ability to provide effective cervical cancer screening education to members of the community, as has been demonstrated in other studies.^33^

A significant implication of the findings of this study is that an effective task-sharing cervical cancer training of CHEWs and CHOs constitutes a robust strategy to fill the human resource gap for diagnosis of early cervical precancerous lesions and early treatment across the continuum of care as prescribed by WHO.^34^ This is particularly important for the first tier of care, the PHCs in LMICs, where nonphysician HCWs often constitute most of the available staff. Task-shifting and task-sharing approaches have been successfully engaged in developing nonphysician-led care of patients with noncommunicable diseases (NCDs) as well as those with HIV/AIDS in several African countries.^31,35,36^ WHO has recognized these innovative human health resource uses as effective and pragmatic approaches to tackling the chronic shortage of HCWs, especially, in resource-poor settings.^37,38^

Our study showed that that trained HCWs (ie, CHEWs and CHOs) can successfully screen individuals in the community for NCDs such as cervical cancer. In 1 year, the CHEWs and CHOs successfully screened 89.3% of the 950 patients for cervical cancer without any reported significant adverse effect. Wider coverage and high uptake of similar services have been demonstrated in other studies.^39-41^ This has improved access to this important preventive health care service at this rural community level, as demonstrated in other similar studies.^42,43^ In the current study, the interrater agreements for the CHEWs and CHOs group and the nurses were 88.1% and 92.3%, respectively, with κ values of 0.76 (good) and 0.84 (very good), respectively, giving an overall concordance rate of 90.5%. This is considered an excellent performance by these less-trained HCWs. In some cancer-screening studies from India and Sri Lanka, the agreement in the clinical diagnosis made by nonphysicians and physicians was 89%.^39,44^ In another study from rural India, the recommendations for drug therapy made by nonphysicians, guided by algorithms, were the same as those made by physicians in > 87% cases of suspected stroke and myocardial infarction.^29^ The validity of this comparison was further improved by the good correlation between the VIA-positive cases and the Papanicolaou test results of 84.2% dysplastic lesions.

However, despite some inherent challenges, such as incessant strike actions in public health institutions and limitations that might exist, like focusing on a single disease in the era of combination prevention approach to diseases, especially those with similar risk factors, some important lessons can be learned from this study. Given the lack of the needed HCWs in LMIC, especially in rural and remote regions, policymakers should consider training and providing guidelines and supportive supervision to the available, less-trained HCWs to screen individuals at the community level and refer them appropriately for additional evaluation and/or treatment. This concept has been demonstrated in a pilot integrated NCD management project in India, in which nonphysician HCWs screen individuals for common NCD risk factors such as tobacco use, physical activity, blood glucose level, blood pressure, weight, height, and body mass index, and refer patients at high risk to physicians at the local health center.^18^ The incorporation of private health institutions in such skill acquisition and services provision will serve as a good bridge for service continuation during industrial closure of public health facilities.^45^

This study provided the evidence for the possibility of cervical cancer screening–specific task-shifting and task-sharing programs in sub-Saharan Africa, a region currently witnessing a rapid expansion of the burden of NCD despite scarce skilled HCWs.^44^ There is need to replicate this concept in more local government areas for a longer period to better understand issues relating to quality of care provided and patient satisfaction. Also, the issues of cost-effectiveness and sustainability, improved availability, and cultural appropriateness need more study.
